# Novel *Acinetobacter baumannii* Bacteriophage Aristophanes Encoding Structural Polysaccharide Deacetylase

**DOI:** 10.3390/v13091688

**Published:** 2021-08-26

**Authors:** Olga Yu. Timoshina, Mikhail M. Shneider, Peter V. Evseev, Anastasia S. Shchurova, Andrey A. Shelenkov, Yulia V. Mikhaylova, Olga S. Sokolova, Anastasia A. Kasimova, Nikolay P. Arbatsky, Andrey S. Dmitrenok, Yuriy A. Knirel, Konstantin A. Miroshnikov, Anastasia V. Popova

**Affiliations:** 1 Shemyakin-Ovchinnikov Institute of Bioorganic Chemistry, Miklukho-Maklaya 16/10, 117997 Moscow, Russia; lalatimosha@gmail.com (O.Y.T.); mikhailshneider@gmail.com (M.M.S.); petevseev@gmail.com (P.V.E.); kmi@bk.ru (K.A.M.); 2 Central Scientific Research Institute of Epidemiology, Novogireevskaya 3a, 111123 Moscow, Russia; shelenkov@cmd.su (A.A.S.); mihailova@cmd.su (Y.V.M.); 3 Moscow Institute of Physics and Technology, National Research University, Institutskiy per. 9, Dolgoprudny, 141700 Moscow, Russia; anastasiya.shchurova@phystech.edu; 4 Biology Department, Lomonosov Moscow State University, Leninskie Gory 1, 119234 Moscow, Russia; sokolova@mail.bio.msu.ru; 5 Biology Department, Shenzhen MSU-BIT University, 1 International University Park Road, Longgang District, Shenzhen 518172, China; 6 N. D. Zelinsky Institute of Organic Chemistry, Russian Academy of Sciences, Leninsky Prospect 47, 119991 Moscow, Russia; nastia-kasimova979797@mail.ru (A.A.K.); Nikolay.Arbatsky@gmail.com (N.P.A.); dmt@ioc.ac.ru (A.S.D.); yknirel@gmail.com (Y.A.K.); 7 State Research Center for Applied Microbiology and Biotechnology, 24 “Quarter A” Territory, Obolensk, City District Serpukhov, 142279 Moscow, Russia

**Keywords:** bacteriophage, *Acinetobacter baumannii*, deacetylase, capsular polysaccharide, capsular type

## Abstract

*Acinetobacter baumannii* appears to be one of the most crucial nosocomial pathogens. A possible component of antimicrobial therapy for infections caused by extremely drug-resistant *A. baumannii* strains may be specific lytic bacteriophages or phage-derived enzymes. In the present study, we observe the biological features, genomic organization, and phage–host interaction strategy of novel virulent bacteriophage Aristophanes isolated on *A. baumannii* strain having K26 capsular polysaccharide structure. According to phylogenetic analysis phage Aristophanes can be classified as a representative of a new distinct genus of the subfamily *Beijerinckvirinae* of the family *Autographiviridae*. This is the first reported *A. baumannii* phage carrying tailspike deacetylase, which caused O-acetylation of one of the K26 sugar residues.

## 1. Introduction

Nowadays, the ESKAPE pathogens (*Enterococcus faecium, Staphylococcus aureus, Klebsiella pneumoniae, Acinetobacter baumannii, Pseudomonas aeruginosa*, and *Enterobacter* sp.) are the leading cause of nosocomial infections worldwide [1]. *A. baumannii* are strictly aerobic, non-fermenting, catalase-positive, oxidase-negative, immobile (non-glutinous, have twitching mobility) Gram-negative coccobacilli with a DNA G+C content of about 39% [2]. In 2017, carbapenem-resistant *A. baumannii* strains was designated by the World Health Organization as pathogens of the “Priority 1: CRITICAL” group within a proposed list of the most significant antibiotic-resistant bacteria for which the development of new antibiotics is extremely necessary [3]. A possible component of antimicrobial therapy of infections caused by drug-resistant *A. baumannii* strains can be specific lytic bacteriophages or enzymes and antibacterial proteins encoded in their genomes.

*A. baumannii* strains can produce a vast variety of capsular polysaccharides (CPSs) which serve as primary receptors for the majority of phages that carry genes encoding polysaccharide-degrading enzymes [4,5,6,7,8,9,10,11]. The structures of *A. baumannii* CPSs are very diverse, comprising above 140 capsular types (K types) have been predicted bioinformatically (J.J. Kenyon, Queensland University of Technology, Brisbane, Australia, personal communication). To date, there are descriptions of depolymerase-carrying bacteriophages specifically infecting *A. baumannii* strains belonging to 15 various K types [4,5,7,8,9,10,11,12].

For now, there was no evidence, that phages infecting *A. baumannii* strains use deacetylation as a phage–host recognition and adsorption strategy. In this study, we have revealed and described the first member of such type of bacterial viruses–phage Aristophanes encoding structural deacetylase.

## 2. Materials and Methods

### 2.1. Bacterial Strains

*A. baumannii* strain KZ1098 used as the bacterial host for phage Aristophanes, was isolated from the respiratory tract of a hospitalized patient in Nur-Sultan, Kazakhstan in 2016. According to SNPT*Ab* database [13] KZ1098 belongs to ST218^Pas^/ST184^Oxf^ sequence types, has K26 capsular type and also carries the *bla_OXA-58-like_* carbapenemase gene. The strain was kindly provided by Drs. Mikhail Edelshtein and Ilya Azizov (Institute of Antimicrobial Chemotherapy, Smolensk State Medical University, Smolensk, Russia).

The host specificity of the phage was tested against a panel of *A. baumannii* strains with confirmed CPS structure belonging to different K types (K1, K2, K3/22, K6, K7, K8, K9, K11, K15, K16, K17, K19, K20, K21, K24, K25, K26, K27, K30, K32, K33, K35, K37, K42, K43, K44, K45, K46, K47, K48, K51, K52, K53, K54, K55, K57, K58, K61, K73, K74, K80, K81, K82, K83, K84, K85, K87, K88, K89, K90, K91, K92, K93, K116, K125, K128) used in our previous work [7]. The strains were kindly provided by the members of research groups from different countries.

### 2.2. Phage Isolation, Propagation, and Purification

Bacteriophage Aristophanes was isolated from a sewage sample (collected in 2018, Moscow, Russia) by an enrichment procedure described previously [7]. The sewage sample was cleared by low-speed centrifugation at 7000× *g* for 15 min to avoid bacterial and mechanical pollution. Then the supernatant was mixed with LB medium and was incubated in presence of growing *A. baumannii* strain KZ1098 as well as other *A. baumannii* strains belonging to different capsular types overnight at 37 °C with shaking, and then chloroform was added. Bacterial debris was pelleted by centrifugation at 7000× *g* for 30 min. Supernatant was subsequently filtered through 1.20 and 0.45-µm-pore-size membrane filters (Millipore, Cork, Ireland). The presence of lytic bacteriophages in the samples was confirmed by a spot test on the lawn of the target strain. The formation of zones of lysis or plaques formation was verified after overnight incubation of plates at 37 °C.

Plaques formed on the lawn of *A. baumannii* strain KZ1098 were picked up and suspended in the SM buffer (50 mM Tris-HCl pH 7.7, 8 mM MgSO_4_, 100 mM NaCl). The resulting SM solution was then titrated to select single plaques to derive pure phage stock.

The phage Aristophanes propagation was executed using liquid culture of *A. baumannii* strain KZ1098 (OD_600_ = 0.3) at the multiplicity of infection (MOI) of 0.1. The incubation was performed at 37 °C until lysis, and then chloroform was added. Bacterial debris was spined down by centrifugation at 7000× *g* for 15 min. Phage particles were precipitated by polyethylene glycol (PEG) 8000 (added to a final concentration of 10% *w*/*v*) and 500 mM NaCl for 24 h at 4 °C. The PEG–phage precipitate was pelleted by centrifugation for 20 min at 9000× *g* at 4 °C, then the phage was resuspended in SM buffer. Phage was cleared of PEG by centrifugation 7000× *g* for 10 min in presence of chloroform (0.5 volume).

The further phage purification was performed by centrifugation in CsCl step gradient at 100,000× *g* for 2 h, opalescent band containing phages was collected, dialyzed against SM buffer, and stored at 4 °C.

### 2.3. Electron Microscopy

Negative-staining transmission electron microscopy [14] was performed to establish the morphology of phage Aristophanes. An aliquot of the purified phage preparation was loaded to the carbon-coated copper grid, subjected to glow-discharge, and subsequently negatively stained with 1% uranyl acetate for 30 s and air-dried. Prepared grids were examined using a JEOL JEM-2100 200 kV transmission electron microscope. Images of negatively stained phage particles were taken with a Gatan Ultrascan 1000XP CCD camera and Gatan Digital Micrograph software. The dimensions were averaged among at least 30 individually measured particles.

### 2.4. Phage Host Specificity Determination

The host specificity of phage Aristophanes was tested against a collection of 56 *A. baumannii* strains belonging to different K types using the double-layer method [15]. For this, 300 µL of *A. baumannii* bacterial cultures grown in LB medium at 37 °C to OD_600_ of 0.3 were mixed with 4 mL of soft agar (LB broth supplemented with 0.6% agarose). The mixtures were plated onto the nutrient agar. Then, the phage suspension (~10^9^ plaque-forming units (PFU) per mL) was spotted on the soft agar lawns and incubated at 37 °C for 18–24 h.

### 2.5. Phage Adsorption and One-Step Growth Experiments

A sample of phage Aristophanes was inoculated in growing culture of *A. baumannii* strain KZ1098 at the approximate MOI of 0.001 and incubated at room temperature. A volume of 100 µL of samples was taken in 0, 1, 3, 5, 8, 10, 15, 20, 25, and 30 min and then mixed with 850 µL of SM buffer with 50 µL of chloroform. After centrifugation, the supernatants were titrated for further determination of unabsorbed phages by the plaque assay method [15] at different time intervals. The procedure was repeated three times. The adsorption constant was calculated according to the study by Adams [15] for a period of 5 min.

For the one-step growth experiments, 20 mL of *A. baumannii* KZ1098 (OD_600_ of 0.3) was harvested by centrifugation (3500× *g*, 10 min, 4 °C) and resuspended in 1 mL LB broth. Bacterial cells were infected with the phage at MOI of 0.01. The bacteriophage was allowed to adsorb for 5 min at 37 °C. Then, the mixture was centrifuged at 13,000× *g* for 2 min to remove unabsorbed phage particles, and the pellet was resuspended in 20 mL of LB broth. Samples were taken at 5- and 10-min intervals for 100 min of incubation at 37 °C and immediately titrated. The procedure was repeated three times.

### 2.6. DNA Isolation, Sequencing, and Phage Genome Annotation

Phage DNA was isolated from concentrated and purified high titer phage stock by standard phenol-chloroform method [16] with previous incubation of sample in 0.5% SDS and 50 µg/mL proteinase K at 65 °C for 20 min.

Genome sequencing was performed on the MiSeq platform using a Nextera DNA library preparation kit (Illumina, San Diego, CA, USA). The generated reads were assembled de novo into single contig using SPAdes v. 3.11.1 [17] with default parameters.

Phage genome was annotated by predicting and validating open reading frames (ORFs) using Prodigal 2.6.1 [18], GeneMarkS 4.3 [19], and Glimmer 3.02 [20]. Identified ORFs were manually curated to ensure fidelity. Functions were assigned to ORFs using a BLAST search on NCBI databases and HHpred [21]. tRNA coding regions were identified with tRNAscan-SE [22] and ARAGORN [23]. Resulting genomes were visualized using Geneious Prime version 2021.1.0. The intergenic genome regions of the phages were searched for promoters with PhagePromoter in the Galaxy framework with threshold 0.65. The position and length of terminal repeats was identified by searching a region with roughly double read depth in comparison to average read depth across the whole genome of the phage. Physical termini were next verified directly by Sanger sequencing with outward-directed primers located inside and outside putative repeats. Predicted proteins were compared using the virulence factor database (VFD) [24].

### 2.7. Nucleotide Sequence Accession Number

Annotated genome of *A. baumannii* phage Aristophanes was deposited to NCBI GenBank under accession number MT783706.

### 2.8. Phylogeny and Taxonomy Studies

Phage genomes for comparison were downloaded from Genbank. Genes of terminase large subunit were extracted from the annotated genomes. Protein alignments were made with MAFFT (L-INS-i algorithm, BLOSUM62 scoring matrix, 1.53 gap open penalty, 0.123 offset value) [25]. The alignments were trimmed manually and with trimAL with gappyout settings. Phylograms were generated based on the amino acid sequences of proteins and their concatenated alignments. Best protein models were found with MEGAX 10.0.5 [26]. Trees were constructed using the maximum likelihood (ML) method with an RAxML program [27,28] and a LG+G protein model [29], and with MrBayes [30,31]. The robustness of the trees was assessed for RAxML by fast bootstrapping (2000) and for MrBayes by the estimation of the average standard deviation in split frequencies.

### 2.9. Genome Comparison, Gene, and Protein Analysis

Average nucleotide identity (ANI) was computed using the OrthoANIu tool, employing USEARCH over BLAST [32] with default settings. The VIRIDIC server was employed for calculating phage intergenomic similarities [33]. Genome comparison was made with Easyfig [34]. Protein remote homology detection and modelling were carried out using HHpred and Phyre2 [35]. Custom BLAST databases were mounted with the BLAST tool.

### 2.10. Cloning, Expression, and Purification of the Recombinant Deacetylase

The DNA sequence of Aristophanes gp41 encoding deacetylase (lacking N-terminal domain of the native phage protein) was PCR amplified using primers 5′-ATAGGATCCGATAGTGCGAGCGTTGCAA and 5′-ATACTCGAGTTATAGATTCTCAAAAATTGGCA and cloned into the pTSL plasmid (GenBank accession KU314761) [36] using BamHI and XhoI restriction sites.

Recombinant protein comprising 167-993aa of the native protein was expressed in *E. coli* B834 (DE3) by induction with 1 mM IPTG at 18 °C overnight. Then the cells were deposited by centrifugation at 4000× *g* for 20 min at 4 °C, resuspended in buffer A (20 mM Tris pH 8.0, 0.4 M NaCl) and sonicated (Virsonic, VirTis, France). The lysate was cleared by centrifugation at 13,000× *g* for 25 min and then loaded into 5 mL Ni2+-NTA Sepharose column (GE Healthcare, Chicago, IL, USA) equilibrated with buffer A. The protein was eluted by a 0–200 mM imidazole step gradient in buffer A. His-tag and SlyD digestion was realized by incubation with TEV-protease overnight with simultaneous dialysis against 10 mM Tris pH 8.0. Final purification was carried out on a 5 mL SourceQ 15 (GE Healthcare, Chicago, IL, USA) using a linear gradient of 0–600 mM NaCl in 20 mM Tris-HCl (pH 8.0).

### 2.11. Phage Infection Inhibition Assay

Phage Aristophanes infection inhibition assay was performed as follows. A titer of 3.5 × 10^6^ plaque forming units (PFU)/mL for the phage was chosen for the competition experiments. *A. baumannii* KZ1098 was grown in LB medium at 37 °C to an OD_600_ of 0.3. Then, Aristophanes_gp41 was added to a 300-μL aliquot of the cell culture to a final concentration of 0.5 mg/mL and incubated for 20 min at 37 °C. Three-hundred-microliter aliquots of the *A. baumannii* host cells with no additives or supplemented with bovine serum albumin (BSA) to a final concentration of 0.5 mg/mL incubated for 20 min at 37 °C served as controls. After the incubation, several dilutions of phage Aristophanes and 4 mL of soft agar were added to the mixtures and plated onto the nutrient agar. The plates were incubated overnight at 37 °C and assayed for the number of lysis plaques. The experiment was performed in triplicate. The GraphPad Prism software (GraphPad Software, Inc., La Jolla, CA, USA) was used for statistical analysis and graphical presentation of the results.

### 2.12. Isolation, Purification, and Deacetylation of the CPS by Recombinant Protein

The *A. baumannii* strain KZ1098 was cultivated in 2TY media overnight at 37 °C. Bacterial cells were harvested by centrifugation (10,000× *g*, 20 min), washed with phosphate-buffered saline, suspended in aqueous 70% acetone, precipitated, and dried on air.

A K26 CPS sample was isolated by phenol-water extraction [37] and purified as described [38].

Purified CPS was solubilized at 20 mM TrisHCl pH 8.0 buffer and recombinant phage protein was added in the ratio of 1/100 *v/v*. The reaction mixture was incubated at 37 °C overnight. CPS processing products were fractionated by gel-permeation chromatography as described previously [7].

### 2.13. NMR Spectroscopy

Samples were deuterium-exchanged by freeze-drying from 99.9% D_2_O. NMR spectra were recorded for solutions in 99.95% D_2_O on a Bruker Avance II 600 MHz spectrometer (Bruker, Germany). Two-dimensional ^1^H-^1^H correlation spectroscopy (COSY), ^1^H ^1^H total correlation spectroscopy (TOCSY), ^1^H-^1^H rotating-frame nuclear Overhauser effect spectroscopy (ROESY), ^1^H-^13^C heteronuclear single-quantum coherence (HSQC), and ^1^H-^13^C heteronuclear multiple-bond correlation (HMBC) experiments were performed using standard Bruker software. Bruker TopSpin 2.1 program was used to acquire and process the NMR data. A spin-lock time of 60 ms and mixing time of 200 ms were used in ^1^H-^1^H TOCSY and ^1^H-^1^H ROESY experiments, respectively. A ^1^H-^13^C HMBC experiment was recorded with a 60 ms delay for evolution of long-range couplings to optimize the spectrum for coupling constant *J*_H,C_ 8 Hz.

## 3. Results

### 3.1. Biology and Morphology of Aristophanes Phage

**Figure 1:**
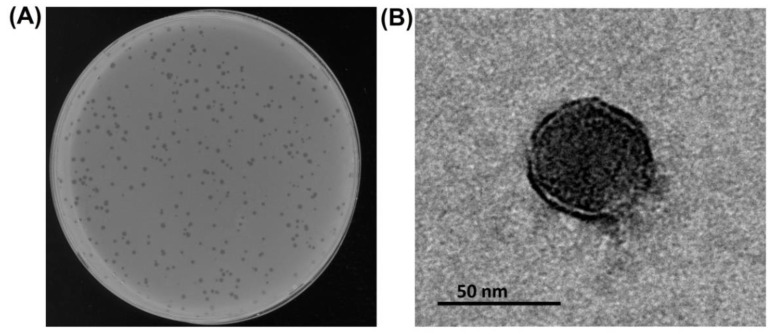
Morphological characteristics of phage Aristophanes. (**A**) Phage plaques on *A. baumannii* KZ1098. (**B**) Transmission electron micrographs of the phage particles. Staining with 1% uranyl acetate. The scale bar is 50 nm.

The *A. baumannii* phage Aristophanes was isolated from a sewage sample and was found to be infectious to *A. baumannii* strain KZ1098 belonging to K26 capsular type. Phage Aristophanes produces small clear plaques without visible halos (Figure 1A) on the host bacterial lawn. Transmission electron microscopy revealed that the phage has isometric icosahedral head with average size of 50 nm in diameter with a short non-contracile tail (Figure 1B).

The host specificity of Aristophanes was tested against a collection of *A. baumannii* strains with biochemically characterized CPS structures belonging to 56 different K types described in our previous study [7]. Phage Aristophanes was found to be highly specific and was capable to infect only *A. baumannii* strain with K26 CPS structure.

**Figure 2:**
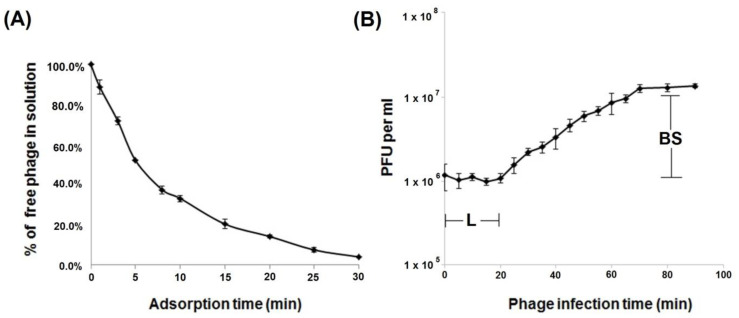
Infection analysis of phage Aristophanes. Adsorption assay (**A**) and one-step growth curve (**B**) of phage Aristophanes on *A. baumannii* KZ1098 with the indication of estimated latent period (L) and burst size (BS). Results are the means and standard deviations from three independent experiments. PFU: plaque-forming units.

The infection process for phage Aristophanes was investigated by estimating the adsorption efficiency and one-step growth experiments. As shown in Figure 2A, almost 50% of phage particles adsorbed to *A. baumannii* KZ1098 cells within 5 min, and more than 80% within 20 min. The phage exhibited adsorption constant of 1.3 × 10^−9^ mL/min for the host strain for a period of 5 min. The latent period for Aristophanes was 20 min (Figure 2B), and the burst size was approximately 15 particles per infected cell.

### 3.2. Phage Genome Organization and Comparison

Phage Aristophanes has linear dsDNA genome of 43,505 bp including flanking 184 bp-long terminal repeats. The G+C content of the Aristophanes genome is 42.5%, close to the G+C content of the *A. baumannii* strains (approximate average value is 39%).

**Figure 3:**
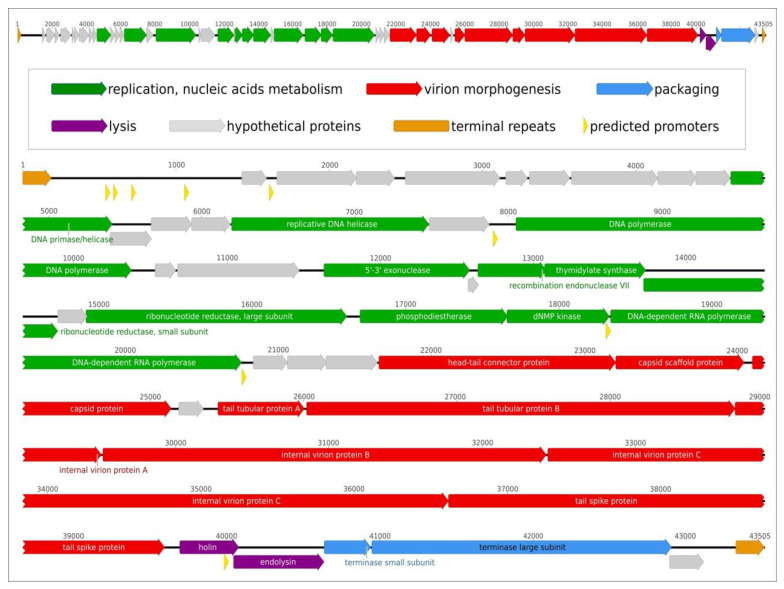
Genetic map of *Acinetobacter* phage Aristophanes. Upper picture: the basic scheme of the genome. Lower picture: the genetic map and putative functions of genes (Table S1).

The genome organization is typical for the phages of family *Autographiviridae*. The Aristophanes genome contains 46 predicted genes encoding 24 proteins with assigned putative functions and 22 hypothetical proteins (Table S1). All the genes are oriented in the same direction. There were no tRNA genes found in the phage genome. The sequence search demonstrated that most of the Aristophanes predicted proteins including the replication and morphogenesis ones are close to the proteins of other *A. baumannii Autographiviridae* bacteriophages. BLAST search with the virulence factor databases (VFDB) did not show a significant similarity with the known virulence factors. The list of predicted promoters includes the promoters anticipating the so-called ‘early region’ genes in the beginning of the genome, the promoters of DNA and RNA polymerases’ genes, the promoters anticipating the blocks of structural and lysis genes. The arrangement of predicted promoters is close to that one of the model phage T7 [39]. The genetic map of phage Aristophanes is shown in Figure 3.

**Figure 4:**
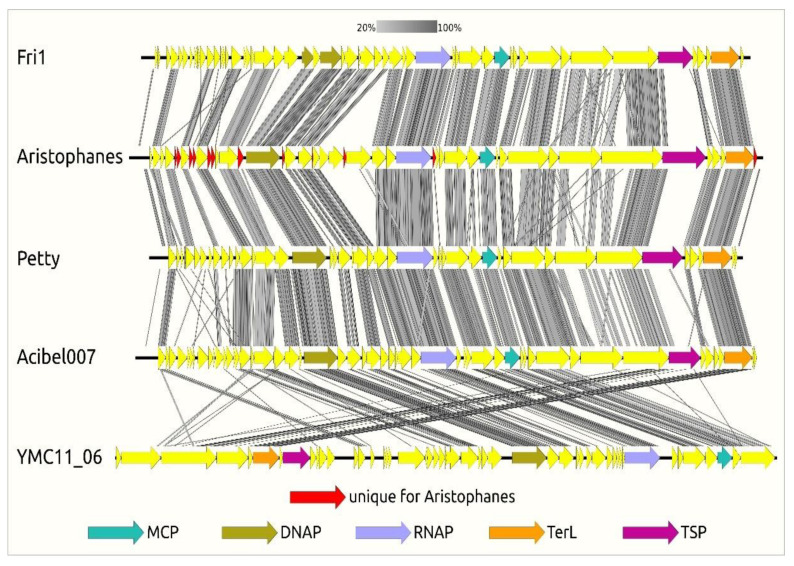
Genome sequence comparison among five *Autographiviridae* genomes exhibiting co-linearity detected by TBLASTX. The percentage of sequence similarity is indicated by the intensity of the grey colour. Vertical blocks between analysed sequences indicate regions with at least 20% similarity. Genes’ names are as follows: MCP, major capsid protein; DNAP, DNA-polymerase; RNAP, DNA-dependent RNA-polymerase; TerL, large subunit of terminase; TSP, tailspike protein; YMC11_06, abbreviated name of *Pseudomonas* phage YMC11/06/C171_PPU_BP.

The intergenomic comparison of four phylogenetically close *A. baumannii* phages, namely Aristophanes, Fri1, Petty, and Acibel007 (belonging to the subfamily *Beijerinckvirinae* of the family *Autographiviridae*), and *Pseudomonas* phage YMC11/06/C171_PPU_BP (belonging to the subfamily *Corkvirinae* of the family *Autographiviridae*) indicates both the overall similarity of the *Autographiviridae* phage genomes and the more prominent similarity of the *Beijerinckvirinae* phage genomes. The differences are located mainly in the early-genes region (Figure 4) encoding products which are most likely involved in inhibiting or redirecting of functionally important host systems [40]. The *Beijerinckvirinae* tailspike proteins (except for the N-termini of the proteins) demonstrate a significant difference in primary structure. The Aristophanes genome contains totally 12 genes unique for this phage (Table S1).

### 3.3. Phage Taxonomy and Phylogeny

The initial full-genome comparison using all complete and draft genome sequences of *Autographiviridae* phages deposited to Genbank was conducted through calculations of average nucleotide identity (ANI) (Table S2). The ANI data testify of relatively distant similarity of the phage Aristophanes with other *Acinetobacter* bacteriophages (ANI of 66.9% or less) belonging to family *Autographiviridae* subfamily *Beijerinckvirinae*. However, ANI values closest to Aristophanes belong to *Acinetobacter* phage vB_AbaP_Acibel007 of genus *Daemvirus* and *Acinetobacter* phage AbKT21phiIII of genus *Friunavirus* (66.9% and 66.4%, correspondingly). This hinders further attribution of Aristophanes to a particular genus. The intergenomic similarity matrix made with the VIRIDIC tool, possibly better estimating the intergenomic similarities [40,41], places the phage Aristophanes near the *Beijerinckvirinae Acinetobacter* phages of the genera of *Daemvirus*, *Pettyvirus*, and *Friunavirus* with the value of 18.8–22% which is far beyond the genus threshold of 70% (Figure S1).

**Figure 5:**
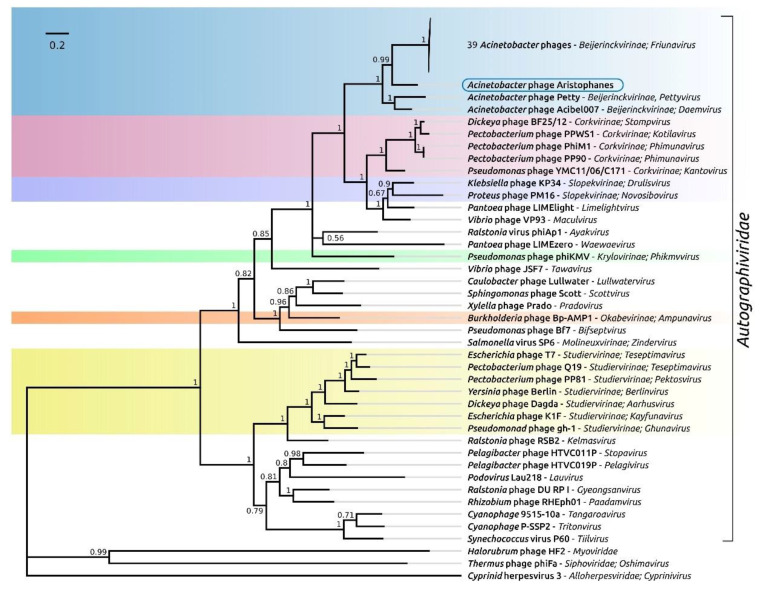
Phylogenetic tree obtained with MrBayes, based on amino acid sequences of terminase large subunit including representatives of all genera of the subfamily *Beijerinckvirinae* of the family *Autographiviridae*. Taxonomic classification is shown to the right of the organism name. Bayesian posterior probabilities are indicated above their branch. The scale bar shows 0.2 estimated substitutions per site and the tree was rooted to Cyprinid herpesvirus 3; 2,100,000 generations sampled every 200 generations, with burn-in length of 100,000 and an average standard deviation of split frequencies of 0.010.

The large subunit of terminase appears to be one of the most conserved proteins encoded in bacteriophage genomes, that makes this protein appropriate for the elucidation of phylogenetic relations [41,42]. The phylogenetic tree, constructed with protein sequences including the representatives of all genera of *Beijerinckvirinae* subfamily places the phage Aristophanes comparatively distantly from other *Beijerinckvirinae* phages infecting *Acinetobacter* spp. This placement is confirmed with a high degree and comparatively significant branch lengths by MrBayes (Figure 5) and the RAxML phylogeny (Figure S2). It seems that the phage Aristophanes can be classified as a representative of an unassigned genus within the *Beijerinckvirinae* subfamily. The Aristophanes phage shares the last common ancestor with the *Acinetobacter* phages Petty belonging to the genus of *Pettyvirus* and Acibel007 belonging to the genus of *Daemvirus*. The *Corkvirinae* and *Slopekvirinae* subfamilies appear to be the closest relatives of the *Beijerinckvirinae* subfamily.

### 3.4. Phage Aristophanes Tailspike Deacetylase

**Figure 6:**
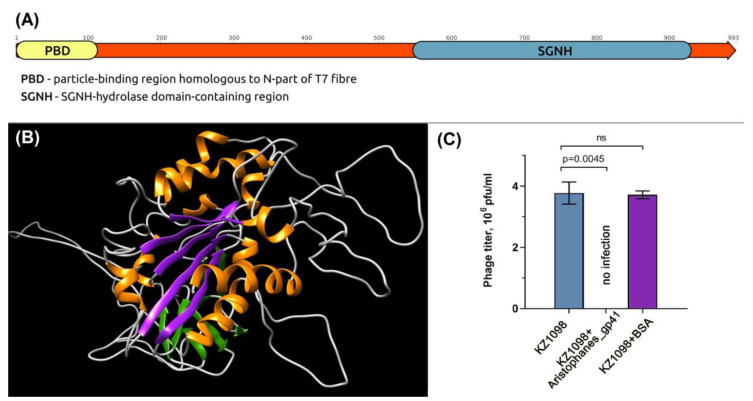
(**A**) Schematic representation of the *Acinetobacter* phage Aristophanes tailspike protein obtained with BLAST search and HMM analysis. (**B**) 3D structure homology modelling of the SGNH-hydrolase domain-containing region of the *Acinetobacter* phage Aristophanes tailspike protein obtained with HHpred. Five parallel β-sheets intrinsic for all SGNH-hydrolases are colored purple, other β-sheets are colored green and the α-helices are colored orange. The model is made using the PDB structure 4K7J of peptidoglycan O-acetylesterase belonging to SGNH-family. (**C**) Phage Aristophanes infection inhibition by Aristophanes_gp41. From left to right, phage titers observed on the bacterial lawns after the treatment of *A. baumannii* KZ1098 cells with phage Aristophanes only, after cell cultures preincubated with purified Aristophanes_gp41, and BSA (as a negative control), followed by phage Aristophanes treatment. P: *p*-value, ns: not significant.

The adsorption apparatus of phage Aristophanes is represented by tailspike protein gp41 (GenBank accession QNO11465) encoded at the end of the structural module of the phage genome (Figure 3 and Figure 4). Aristophanes_gp41 is 993-amino-acid protein with predicted molecular weight of 108.9 kDa. The bioinformatic analysis conducted with BLAST and HMM searches indicate the modular structure of this protein (Figure 6A). The N-terminal part of gp41 possesses the typical phage particle binding structure. The C-part of the protein appears to share structural features with the SGNH/GDSL-hydrolases, including the five parallel *β*-sheets intrinsic for all SGNH-hydrolases (Figure 6A,B).

BLASTp analysis revealed that the closest homolog of Aristophanes_gp41 is a hypothetical protein of *Acinetobacter pittii* (WP_130128013, the coverage obtained to an E-value of 0.0 was 78% with an identity of 81.39%). However, there was no homology between N-terminal parts of these proteins. HHpred analysis shows that the structure of Aristophanes_gp41 N-terminal domain is similar to the structures of N-terminal domains of *A. baumannii* phage phiAB6 tailspike (HHpred probability >98%) and mature bacteriophage T7 tail fiber protein gp17 with E-value of 0.00012. The C-terminal half (548–991aa) of the Aristophanes_gp41 structurally corresponds to GDSL-like lipase/acylhydrolase family protein of *Neisseria meningitidis* (HHpred probability >97%).

Recombinant Aristophanes_gp41 (167-993aa), lacking N-terminal part, was expressed in *E. coli* and purified as described. On the bacterial lawn of *A. baumannii* KZ1098 the protein forms very weak opaque halo (data not shown). Competition experiments have been performed to show that Aristophanes_gp41 plays a key role in the initial stage of the interaction between the phage and the bacterial host (Figure 6C). *A. baumannii* KZ1098 bacterial cultures preincubated with purified Aristophanes_gp41 and with BSA (as a negative control) were mixed with several phage Aristophanes dilutions and plated onto the nutrient agar. After overnight incubation, the phage plaques were measured. It was shown that coincubation of the cells with gp41 completely blocked the phage Aristophanes infection, whereas coincubation of host bacterial cells with BSA showed no significant difference in phage titers.

### 3.5. Deacetylation of the K26 CPS by Aristophanes_gp41 Recombinant Protein

**Figure 7:**
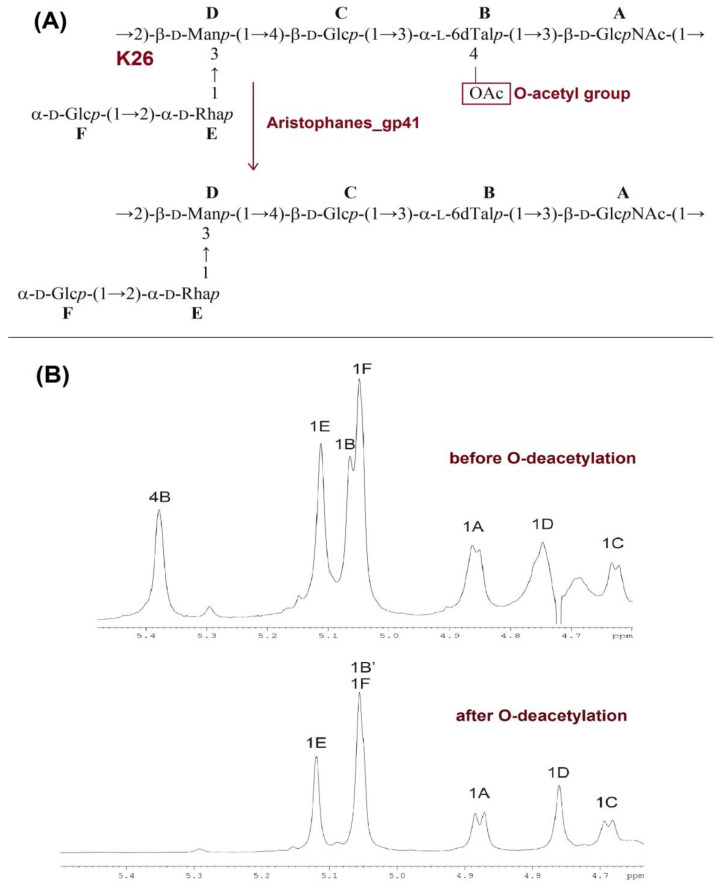
(**A**) Structures of the CPS of *A. baumannii* KZ1098 before and after O-deacetylation. (**B**) Parts of ^1^H NMR spectra of the *A. baumannii* KZ1098 CPS before and after O-deacetylation (MPS).

Considering that bacteriophage Aristophanes forms plaques without visible halos, unlike other *A. baumannii* phages of the family *Autographiviridae*, and the purified protein Aristophanes_gp41 forms very weak halo on the bacterial lawn of the host strain in comparison with previously described tailspike depolymerases [4,5,7,8,9,10,11] we assumed that the mechanism of Aristophanes_gp41 interaction with K26 capsular polysaccharides is not based on total cleavage of CPS to monomers or oligomers of the K unit. To elucidate the exact mechanism of action of Aristophanes_gp41, K26 CPS of *A. baumannii* KZ1098 was treated with the purified protein, and the resultant modified polysaccharide (MPS) was studied by NMR spectroscopy. The ^1^H and ^13^C NMR spectra of the MPS were fully assigned using two-dimensional shift-correlated experiments (^1^H-^1^H COSY, ^1^H-^1^H TOCSY, and ^1^H-^13^C HSQC). Based on these data combined with ^1^H,^1^H and ^13^C,^1^H correlation patterns revealed by two-dimensional ^1^H-^1^H ROESY and ^1^H-^13^C HMBC experiments, the structure of the MPS was established (Figure 7A).

Treatment of the K26 CPS with Aristophanes_gp41 caused no changes to the NMR spectral data of the constituent sugar residues except for 6-deoxytalose (6dTal) (unit B). An unusual low-field position of the H-4 signal of 6dTal at δ_H_ 5.38 was observed in the CPS and accounted for by 4-O-acetylation of this sugar residue. Treatment with the phage tailspike protein caused an upfield shift of this signal to δ_H_ 3.93 (Figure 7B). This displacement was evidently due to elimination of a deshielding effect of an O-acetyl group that was, therefore, located at position 4 of unit B in the initial CPS (Figure 7B). Otherwise, the ^1^H and ^13^C NMR spectra of the CPS before and after O-deacetylation did not differ essentially, and hence, O-deacetylation caused no other structural changes.

To sum up, Aristophanes_gp41 was found to be deacetylase which caused O-acetylation of one of the K26 sugar residues.

## 4. Discussion

In this paper, we investigate phage Aristophanes isolated on the bacterial lawn of *A. baumannii* strain with a K26 CPS structure. This phage was shown to be rather unique among all previously described *A. baumannii* viruses that have been combined into the new family *Autographiviridae* created in 2019 [43]. According to the results of the genomic and phylogenetic analysis, the phage belongs to the subfamily *Beijerinckvirinae* comprising the *Acinetobacter* phages of the genera *Friunavirus*, *Pettyvirus*, and *Daemvirus*. However, while sharing the common features of the above phage genera (e.g., the genomic structure and homologous proteins), phage Aristophanes substantially differs from other *Beijerinckvirinae* phages in the overall nucleotide similarity. About a quarter of the Aristophanes predicted proteins can be considered as unique ones. These differences in nucleotide composition together with the phylogenetic analysis of the large subunit of terminase suggest the assignment of the phage to a new separate genus of the subfamily *Beijerinckvirinae* of the family *Autographiviridae*.

Another distinctive characteristic of Aristophanes is that the phage produces small clear plaques without visible halos on the host bacterial lawn unlike all previously described *A. baumannii* viruses of the family *Autographiviridae*. It was shown that the other reported phages of the family form plaques with haloes [6,7,8,10] indicating the presence of structural depolymerases or tailspike proteins with CPS-degrading activity. When infecting bacteria covered with a thick layer of surface polysaccharide phages often use O-polysaccharide or CPS as a primary receptor. Further action usually involves local degradation of polysaccharide using an enzymatic domain within a tailspike protein. A broad variety of such phage structural polysaccharide depolymerases is studied (reviewed in [44,45]). Tailspike of phage Aristophanes (Aristophanes_gp41) was formed by a single protein encoded by the gene located at the end of structural module of the phage genome like in other *A. baumannii* phages of the family *Autographiviridae*. The absence of halos surrounding the phage plaques suggested that Aristophanes_gp41 acts in a different way than depolymerases degrading glycosidic linkages in CPSs of host strains. Whereas all reported depolymerases with characterized mechanisms of action encoded in *Autographiviridae A. baumannii* phage genomes were shown to be specific glycosidases [5,7,46], Aristophanes_gp41 was found to be a deacetylase which caused O-acetylation of one of the host CPS sugar residues. Since the NMR spectra of the CPS before and after O-deacetylation by Aristophanes_gp41 did not differ essentially, O-deacetylation caused no significant CPS structural changes. For this reason, most likely, halos (or areas with decapsulated bacterial cells) are not formed around the phage plaques.

Some phages infecting *Enterobacterales* were shown to use a strategy of deacetylation of complex carbohydrates [47,48], but this mechanism was never observed for *Acinetobacter* phages previously. It is not clear how the removal of acetyl groups of carbohydrates promotes the spatial approaching of the phage to bacterial cell wall. However, a crucial role of Aristophanes’ deacetylase in the initial step of the phage–bacterial cell interaction was shown in a set of competition experiments. The phage was unable to infect K26 *A. baumannii* cells pretreated with purified Aristophanes_gp41 due to the modification of CPS. The presented results indicate that phages may use deacetylation of outer polysaccharides as a universal mechanism of reception.

Discussing the therapeutic potential of phage Aristophanes it is worth to note that the phage specificity to a single capsular type is typical for *Acinetobacter* phages [7]. Therefore, the elucidation of the applicability of the particular phage species for phage therapy depends on the prevalence of the pathogen with the targeted CPS in each treatment. Despite CPS is considered as an important factor in *A. baumannii* virulence [49] and biofilm formation [50], no convenient method to determine the K type exists up to date, and no information on abundance of *A. baumannii* strains with different K types in clinical practice is available. An acceptable solution could be a creation of a phage collection comprising characterized phages infectious to all known K types of *A. baumannii*. Phage Aristophanes selectively infecting K26 *A. baumannii* cells using an identified reception mechanism at least could be an integral part of such phage panel.

## 5. Conclusions

In this study, we present a characterization of the novel *A. baumannii* phage Aristophanes which can be classified as a representative of a new distinct genus of the *Beijerinckvirinae* subfamily of the *Autographiviridae* family according to phylogenetic analysis. This is the first reported *A. baumannii* virulent phage carrying tailspike deacetylase. Aristophanes_gp41 was found to play a key role in the initial stage of the phage–host interaction. The ^1^H and ^13^C NMR spectra of the K26 CPS of *A. baumannii* KZ1098 before and after cleavage by recombinant deacetylase did not differ essentially (there is an elimination of an O-acetyl group), so O-deacetylation caused no deep structural changes. Detailed characterization of novel *A. baumannii* lytic phages and their viral–bacterial host interactions provide us better understanding of applying phages in personalized medicine.

## Data Availability

Annotated genome of *A. baumannii* phage Aristophanes was deposited to GenBank under accession number MT783706.

## Supplementary Materials

The following are available online at https://www.mdpi.com/article/10.3390/v13091688/s1, Table S1. Functional assignments of *Acinetobacter* phage Aristophanes genes. Table S2. Average nucleotide identity (ANI) between *Acinetobacter* phage Aristophanes and all phage genomes deposited in the NCBI GenBank (calculated with orthoANIu, threshold 0.5). Figure S1. VIRIDIC generated heatmap of 85 representatives of all genera of the family *Autographiviridae*. Figure S2. Best-scoring tree found by maximum likelihood (ML) search with RAxML based on amino acid sequences of terminase large subunit including representatives of all genera of the subfamily *Beijerinckvirinae* of the family *Autographiviridae*.
