# Supplementary material for: Novel Acinetobacter baumannii Bacteriophage Aristophanes Encoding Structural Polysaccharide Deacetylase
Source: Viruses. 2021 Aug 26;13(9):1688. doi: 10.3390/v13091688 (PMC8471582; doi:10.3390/v13091688)
Supplement: Supplementary file 1 [file viruses-13-01688-s001.zip › viruses-1337107-supplementary.pdf]

Supplementary Materials

Table S1. Functional assignments of *Acinetobacter* phage Aristophanes genes.

№	product	Start	End	Length	Direction	locus_tag
1	hypothetical protein	1429	1590	162	forward	Aristophanes_00001
2	hypothetical protein	1658	2176	519	forward	Aristophanes_00002
3	hypothetical protein	2176	2424	249	forward	Aristophanes_00003
4	hypothetical protein	2496	3110	615	forward	Aristophanes_00004
5	hypothetical protein	3151	3291	141	forward	Aristophanes_00005
6	hypothetical protein	3304	3567	264	forward	Aristophanes_00006
7	hypothetical protein	3577	4140	564	forward	Aristophanes_00007
8	hypothetical protein	4142	4393	252	forward	Aristophanes_00008
9	hypothetical protein	4390	4614	225	forward	Aristophanes_00009
10	DNA primase/helicase	4618	5418	801	forward	Aristophanes_00010
11	hypothetical protein	5403	5675	273	forward	Aristophanes_00011
12	hypothetical protein	5672	5938	267	forward	Aristophanes_00012
13	hypothetical protein	5935	6189	255	forward	Aristophanes_00013
14	replicative DNA helicase	6196	7482	1287	forward	Aristophanes_00014
15	hypothetical protein	7485	7877	393	forward	Aristophanes_00015
16	putative DNA polymerase	8051	10378	2328	forward	Aristophanes_00016
17	hypothetical protein	10532	10666	135	forward	Aristophanes_00017
18	hypothetical protein	10677	11471	795	forward	Aristophanes_00018
19	putative 5'-3' exonuclease	11632	12582	951	forward	Aristophanes_00019
20	hypothetical protein	12572	12640	69	forward	Aristophanes_00020
21	recombination endonuclease VII	12637	13080	444	forward	Aristophanes_00021
22	thymidylate synthase	13073	13729	657	forward	Aristophanes_00022
23	ribonucleotide reductase, small subunit	13716	14729	1014	forward	Aristophanes_00023
24	hypothetical protein	14731	14922	192	forward	Aristophanes_00024
25	ribonucleotide reductase, large subunit	14915	16615	1701	forward	Aristophanes_00025
26	putative phosphodiestherase	16702	17664	963	forward	Aristophanes_00026
27	dNMP kinase	17661	18326	666	forward	Aristophanes_00027
28	putative DNA-dependent RNA polymerase	18335	20761	2427	forward	Aristophanes_00028
29	hypothetical protein	20841	21062	222	forward	Aristophanes_00029
30	hypothetical protein	21063	21317	255	forward	Aristophanes_00030
31	hypothetical protein	21314	21649	336	forward	Aristophanes_00031
32	putative head-tail connector protein	21659	23203	1545	forward	Aristophanes_00032
33	putative capsid scaffold protein	23203	24039	837	forward	Aristophanes_00033
34	putative capsid protein	24094	25137	1044	forward	Aristophanes_00034
35	hypothetical protein	25187	25348	162	forward	Aristophanes_00035
36	tail tubular protein A	25443	26006	564	forward	Aristophanes_00036
37	tail tubular protein B	26022	28814	2793	forward	Aristophanes_00037
38	internal virion protein A	28815	29516	702	forward	Aristophanes_00038

39	putative internal virion protein B	29527	32418	2892	forward	Aristophanes_00039
40	putative internal virion protein C	32429	36613	4185	forward	Aristophanes_00040
41	tailspike protein	36615	39596	2982	forward	Aristophanes_00042
42	putative holin	39697	40080	384	forward	Aristophanes_00043
43	endolysin	40049	40636	588	forward	Aristophanes_00044
44	putative DNA maturase subunit A	40639	40941	303	forward	Aristophanes_00045
45	putative DNA maturase subunit B	40950	42902	1953	forward	Aristophanes_00046
46	hypothetical protein	42889	43113	225	forward	Aristophanes_00047

Figure S1. VIRIDIC generated heatmap of 85 representatives of all genera of the family *Autographiviridae*.

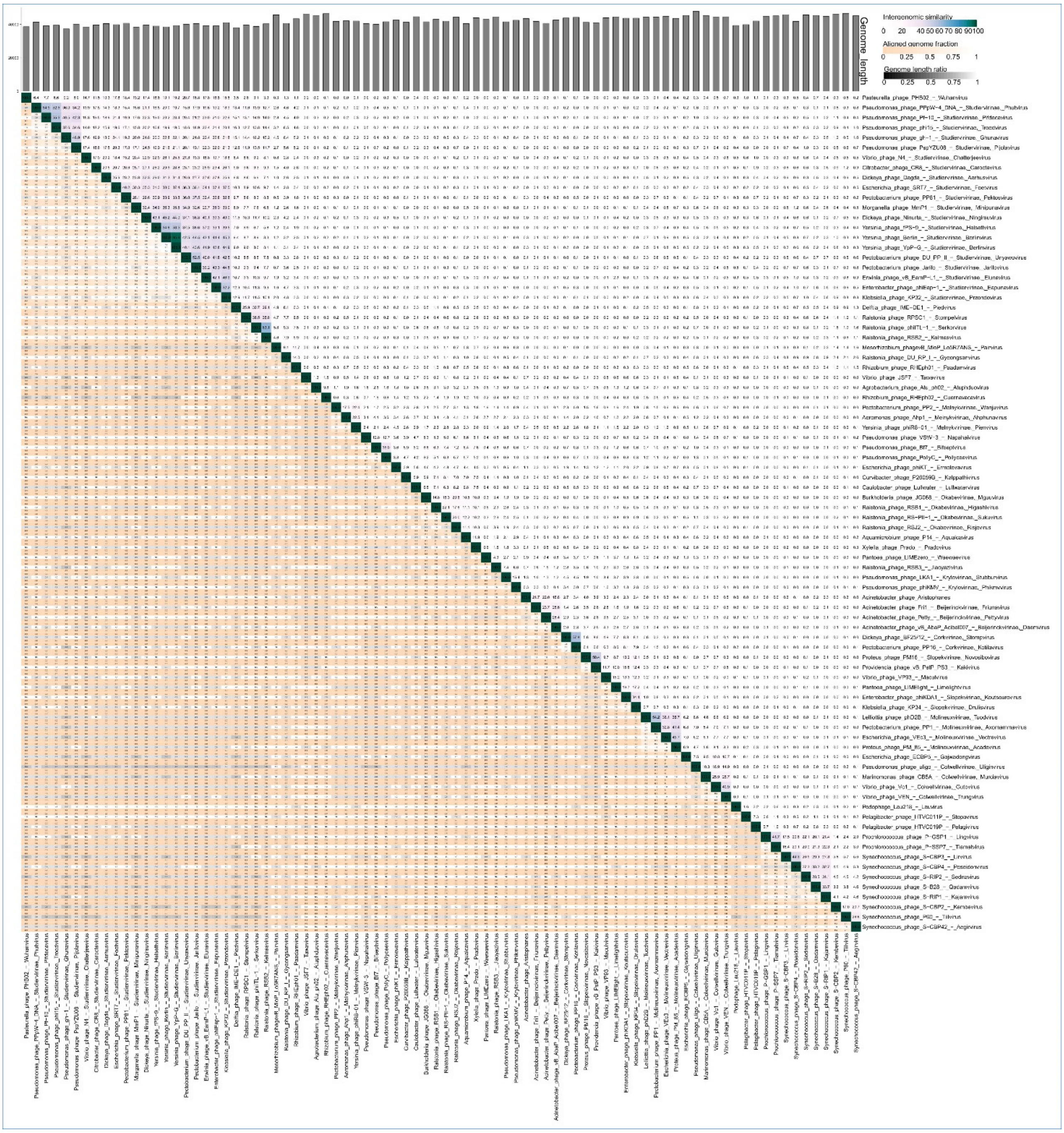

The heatmap incorporates intergenomic similarity values (right half) and alignment indicators (left half and top annotation). In the right half, the colour coding indicates the clustering of the phage genomes based on intergenomic similarity. The numbers represent the similarity values for each genome pair, rounded to the first decimal. In the left half, three indicator values are represented for each genome pair, from top to bottom: aligned fraction genome 1 (for the genome found in this row), genome length ratio (for the two genomes in this pair) and aligned fraction genome 2 (for the genome found in this column).

Figure S2. Best-scoring tree found by maximum likelihood (ML) search with RAxML based on amino acid sequences of terminase large subunit including representatives of all genera of the subfamily *Beijerinckvirinae* of the family *Autographiviridae*.

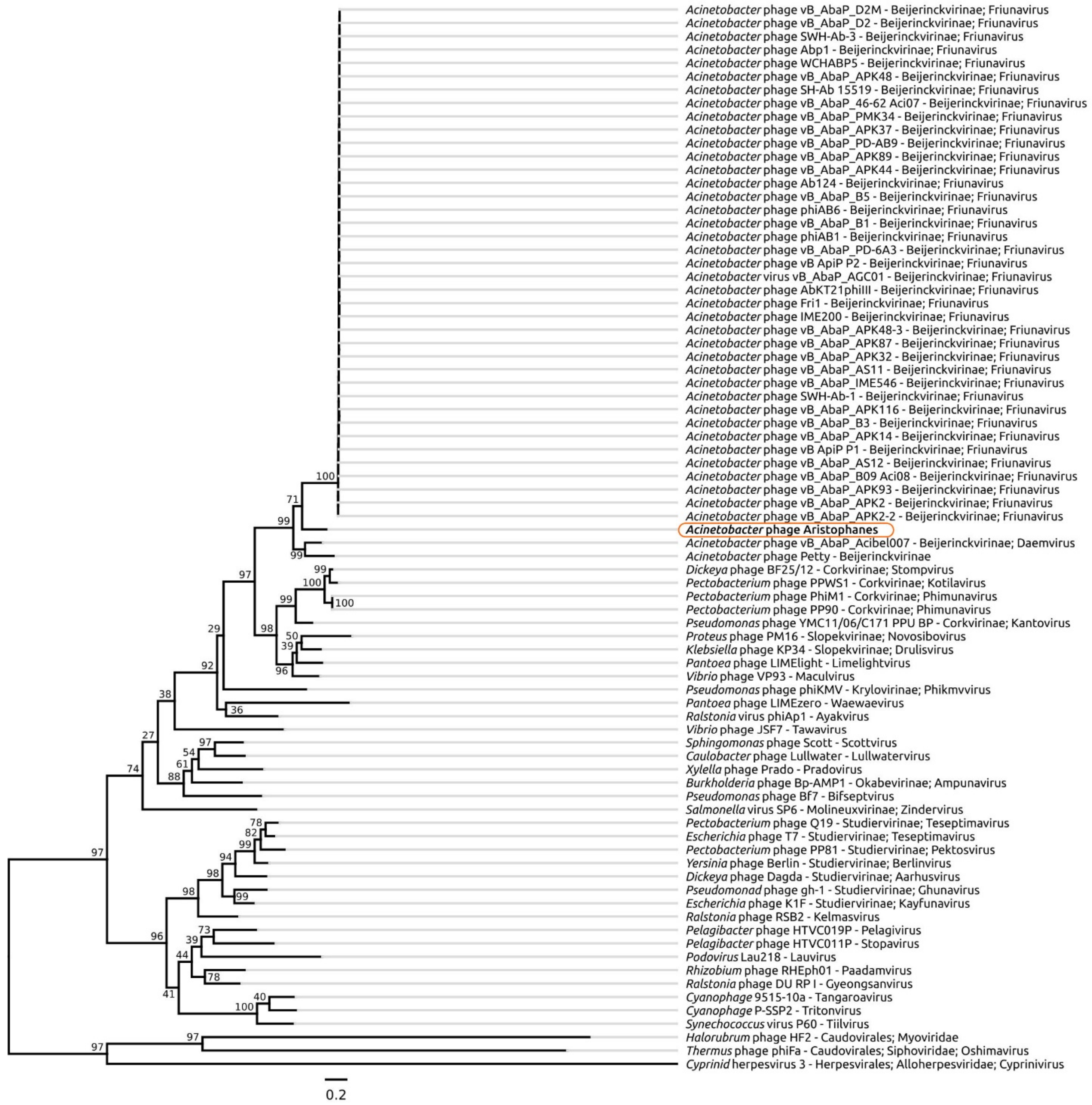

Taxonomic classification is shown to the right of the organism name. Bootstrap support values are shown above their branch as a percentage of 2000 replicates. The scale bar shows 0.2 estimated substitutions per site and the tree was rooted to *Cyprinid* herpesvirus 3, *Halorubrum* phage HF2 and *Thermus* phage phiFa.

Table S2. Average nucleotide identity (ANI) between *Acinetobacter* phage Aristophanes and all phage genomes deposited in the NCBI GenBank (calculated with orthoANIu, threshold 0.5).

NCBI Accession	Organism	ANI	NCBI Taxonomy
MT783706	*Acinetobacter* phage Aristophanes	100	*Autographiviridae*; *Beijerinckvirinae*; unclassified *Beijerinckvirinae*
KJ473423	*Acinetobacter* phage vB_AbaP_Acibel007	66,9	*Autographiviridae*; *Beijerinckvirinae*; *Daemvirus*; *Acinetobacter* virus Acibel007
MK278859	*Acinetobacter* phage AbKT21phiIII	66,4214	*Autographiviridae*; *Beijerinckvirinae*; *Friunavirus*; *Acinetobacter* virus AbKT21III
MN294712	*Acinetobacter* phage vB_AbaP_APK48	65,8857	*Autographiviridae*; *Beijerinckvirinae*; *Friunavirus*; unclassified *Friunavirus*
KT388102	*Acinetobacter* phage vB_AbaP_PD-6A3	65,8375	*Autographiviridae*; *Beijerinckvirinae*; *Friunavirus*
MW366784	*Klebsiella* phage Paty	65,6143	*Autographiviridae*; *Beijerinckvirinae*; *Friunavirus*; unclassified *Friunavirus*
KR149290	*Acinetobacter* phage Fri1	65,6125	*Autographiviridae*; *Beijerinckvirinae*; *Friunavirus*
MN856111	*Myoviridae* sp.	65,1833	*Myoviridae*
KY268295	*Acinetobacter* phage vB_AbaP_AS12	65,1667	*Autographiviridae*; *Beijerinckvirinae*; *Friunavirus*; *Acinetobacter* virus AS12
MF033348	*Acinetobacter* phage vB_AbaP_B3	64,9625	*Autographiviridae*; *Beijerinckvirinae*; *Friunavirus*; *Acinetobacter* virus B3
MN651570	*Acinetobacter* phage vB_AbaP_APK89	64,7889	*Autographiviridae*; *Beijerinckvirinae*; *Friunavirus*; unclassified *Friunavirus*
MG599035	*Acinetobacter* phage SWH-Ab-3	64,7778	*Autographiviridae*; *Beijerinckvirinae*; *Friunavirus*; *Acinetobacter* virus SWHAb3
KY888680	*Acinetobacter* phage WCHABP5	64,6667	*Autographiviridae*; *Beijerinckvirinae*; Friunavirus; *Acinetobacter* virus WCHABP5
MW056501	*Acinetobacter* phage vB_AbaA_fBenAci001	64,6222	*Autographiviridae*; *Beijerinckvirinae*; *Friunavirus*; unclassified *Friunavirus*
MN604239	*Acinetobacter* phage vB_AbaP_APK87	64,6	*Autographiviridae*; *Beijerinckvirinae*; *Friunavirus*; unclassified *Friunavirus*
MK257722	*Acinetobacter* phage vB_AbaP_APK32	64,52	*Autographiviridae*; *Beijerinckvirinae*; *Friunavirus*; unclassified *Friunavirus*
MF033347	*Acinetobacter* phage vB_AbaP_B1	64,45	*Autographiviridae*; *Beijerinckvirinae*; *Friunavirus*; *Acinetobacter* virus B1

KT804908	*Acinetobacter* phage IME200	64,3556	*Autographiviridae*; *Beijerinckvirinae*; *Friunavirus*
MN433707	*Acinetobacter* phage vB_AbaP_PMK34	64,3444	*Autographiviridae*; *Beijerinckvirinae*; *Friunavirus*; unclassified *Friunavirus*
MN212906	*Acinetobacter* phage vB_AbaP_D2M	64,3167	*Autographiviridae*; *Beijerinckvirinae*; *Friunavirus*; unclassified *Friunavirus*
MN807295	*Acinetobacter* phage vB_AbaP_APK116	64,2625	*Autographiviridae*; *Beijerinckvirinae*; *Friunavirus*; unclassified *Friunavirus*
HQ186308	*Acinetobacter* phage phiAB1	64,2222	*Autographiviridae*; *Beijerinckvirinae*; *Friunavirus*
KT388103	*Acinetobacter* phage vB_AbaP_PD-AB9	64,2	*Autographiviridae*; *Beijerinckvirinae*; *Friunavirus*
JX658790	*Acinetobacter* phage Abp1	64,19	*Autographiviridae*; *Beijerinckvirinae*; *Friunavirus*
MN614471	*Acinetobacter* phage vB_AbaP_APK48-3	64,1667	*Autographiviridae*; *Beijerinckvirinae*; *Friunavirus*; unclassified *Friunavirus*
KY082667	*Acinetobacter* phage SH-Ab 15519	64,08	*Autographiviridae*; *Beijerinckvirinae*; *Friunavirus*; *Acinetobacter* virus SH-Ab 15519
MK474470	*Vibrio* phage vB_VpaS_OWB	64	*Autographiviridae*; *Maculvirus*; *Vibrio* virus OWB
MN974282	*Vibrio* phage OWB	64	*Autographiviridae*; *Maculvirus*; *Vibrio* virus OWB
MF033349	*Acinetobacter* phage vB_AbaP_B5	63,9875	*Autographiviridae*; *Beijerinckvirinae*; *Friunavirus*; *Acintetobacter* virus B5
MK089780	*Acinetobacter* phage vB_AbaP_APK14	63,9556	*Autographiviridae*; *Beijerinckvirinae*; *Friunavirus*; unclassified *Friunavirus*
MT741944	*Acinetobacter* phage vB_AbaP_APK81	63,9556	*Autographiviridae*; *Beijerinckvirinae*; *Friunavirus*; unclassified *Friunavirus*
MW366783	*Acinetobacter* phage Pipo	63,8769	*Autographiviridae*; *Beijerinckvirinae*; *Friunavirus*; unclassified *Friunavirus*
KF669658	*Acinetobacter* phage Presley	63,7	*Podoviridae*
MT633129	*Acinetobacter* phage Ab124	63,7	*Autographiviridae*; *Beijerinckvirinae*; *Friunavirus*; unclassified *Friunavirus*
MF033350	*Acinetobacter* phage vB_ApiP_P1	63,6444	*Autographiviridae*; *Beijerinckvirinae*; *Friunavirus*; *Acinetobacter* virus P1
MF754112	*Vibrio* phage vB_VpaP_KF2	63,6	*Autographiviridae*; *Maculvirus*; *Vibrio* virus KF2
MH763831	*Acinetobacter* phage vB_AbaP_B09_Aci08	63,4692	*Autographiviridae*; *Beijerinckvirinae*; *Friunavirus*; *Acinetobacter* virus Aci08
MG459218	*Acinetobacter* phage SWH-Ab-1	63,4625	*Autographiviridae*; *Beijerinckvirinae*; *Friunavirus*; *Acinetobacter* virus SWHAb1
KC311669	*Acinetobacter* phage AB3	63,4571	*Autographiviridae*; *Beijerinckvirinae*; *Friunavirus*
KT339321	*Acinetobacter* phage phiAB6	63,4364	*Autographiviridae*; *Beijerinckvirinae*; *Friunavirus*; *Acinetobacter* virus

			phiAB6
MK257720	*Acinetobacter* phage vB_AbaP_APK2-2	63,3778	*Autographiviridae*; *Beijerinckvirinae*; *Friunavirus*; unclassified *Friunavirus*
MW056503	*Acinetobacter* phage vB_AbaA_fBenAci003	63,3667	*Autographiviridae*; *Beijerinckvirinae*; *Friunavirus*; unclassified *Friunavirus*
MK257719	*Acinetobacter* phage vB_AbaP_APK2	63,3556	*Autographiviridae*; *Beijerinckvirinae*; *Friunavirus*; unclassified *Friunavirus*
MN395291	*Acinetobacter* phage vB_AbaP_IME546	63,3556	*Autographiviridae*; *Beijerinckvirinae*; *Friunavirus*; unclassified *Friunavirus*
MN604238	*Acinetobacter* phage vB_AbaP_APK44	63,3545	*Autographiviridae*; *Beijerinckvirinae*; *Friunavirus*; unclassified *Friunavirus*
MH042230	*Acinetobacter* phage vB_AbaP_D2	63,3125	*Autographiviridae*; *Beijerinckvirinae*; *Friunavirus*; *Acinetobacter* virus D2
MT263719	*Acinetobacter* virus vB_AbaP_AGC01	63,28	*Autographiviridae*; *Beijerinckvirinae*; *Friunavirus*; unclassified *Friunavirus*
MW331544	*Vibrio* phage vB_Vc_SrVc2	63,2	*Autographiviridae*; *Maculvirus*; unclassified *Maculvirus*
MK257721	*Acinetobacter* phage vB_AbaP_APK93	63,1846	*Autographiviridae*; *Beijerinckvirinae*; *Friunavirus*; unclassified *Friunavirus*
MF754111	*Vibrio* phage vB_VpaP_KF1	63,1	*Autographiviridae*; *Maculvirus*; *Vibrio* virus KF1
MG575418	*Proteus* phage vB_PmiP_RS1pmA	63,1	*Autographiviridae*; *Slopekvirinae*; *Novosibovirus*; unclassified *Novosibovirus*
MT178448	*Vibrio* phage vB_VpP_FE11	63,1	*Autographiviridae*; *Maculvirus*; unclassified *Maculvirus*
MK257723	*Acinetobacter* phage vB_AbaP_APK37	62,9727	*Autographiviridae*; *Beijerinckvirinae*; *Friunavirus*; unclassified *Friunavirus*
MF033351	*Acinetobacter* phage vB_ApiP_P2	62,9333	*Autographiviridae*; *Beijerinckvirinae*; *Friunavirus*; *Acinetobacter* virus P2
LN610577	*Pseudomonas* phage vB_PaeS_PAO1_Ab18	62,9	*Siphoviridae*; Abidjanvirus
KY268296	*Acinetobacter* phage vB_AbaP_AS11	62,8333	*Autographiviridae*; *Beijerinckvirinae*; *Friunavirus*; *Acinetobacter* virus AS11
LN610585	*Pseudomonas* phage vB_PaeS_PAO1_Ab20	62,8	*Siphoviridae*; *Abidjanvirus*
HE956710	*Yersinia* phage phi80-18	62,5	*Autographiviridae*; *Melnykvirinae*; *Pokrovskaiavirus*; *Yersinia* virus Phi80-18
KM819694	*Proteus* phage PM 75	62,5	*Autographiviridae*; *Slopekvirinae*; *Novosibovirus*; *Proteus* virus PM75

MH800200	*Acinetobacter* phage vB_AbaP_46-62_Aci07	62,4889	*Autographiviridae*; *Beijerinckvirinae*; *Friunavirus*; *Acinetobacter* virus Aci07
MW056502	*Acinetobacter* phage vB_AbaA_fBenAci002	62,43	*Autographiviridae*; *Beijerinckvirinae*; *Friunavirus*; unclassified *Friunavirus*
MG878892	*Salmonella* phage vB_SpuP_Spp16	62,4	*Autographiviridae*; *Melnykvirinae*; *Panjvirus*; *Salmonella* virus Spp16
KF669656	*Acinetobacter* phage Petty	62,3143	*Autographiviridae*; *Beijerinckvirinae*; *Pettyvirus*; *Acinetobacter* virus Petty
MN061582	*Acinetobacter* phage vB_AbaP_IME546	62,2812	*Autographiviridae*; *Beijerinckvirinae*; *Friunavirus*; unclassified *Friunavirus*
KY318515	*Yersinia* phage fHe-Yen3-01	62,1	*Autographiviridae*; *Melnykvirinae*; *Pokrovskaiavirus*; *Yersinia* virus fHeYen301
MN497414	*Vibrio* phage vB_VhaP_VH-5	61,9125	*Autographiviridae*; unclassified *Autographiviridae*
MG575419	*Proteus* phage vB_PmiP_RS8pmA	61,2	*Autographiviridae*; *Slopekvirinae*; *Novosibovirus*; unclassified *Novosibovirus*
KY385423	*Klebsiella* phage vB_KpnP_KpV74	61,1	*Autographiviridae*; *Slopekvirinae*; *Drulisvirus*; *Klebsiella* virus KpV74
MT459144	*Vibrio* phage Dax	61,1	*Demerecviridae*; *Ermolyevavirinae*; *Cetovirus*; unclassified *Cetovirus*
JX290549	*Pectobacterium* phage PhiM1	60,9	*Autographiviridae*; *Corkvirinae*; *Phimunavirus*; Pectobacterium virus fM1
MH807814	*Pectobacterium* phage Lelidair	60,9	*Autographiviridae*; *Corkvirinae*; *Phimunavirus*; *Pectobacterium* virus Lelidair
MH807816	*Pectobacterium* phage Momine	60,9	*Autographiviridae*; *Corkvirinae*; *Phimunavirus*; unclassified *Phimunavirus*
MK095200	*Pectobacterium* phage Clickz_B8	60,9	*Autographiviridae*; *Corkvirinae*; *Phimunavirus*; unclassified *Phimunavirus*
MK095203	*Pectobacterium* phage Koot	60,9	*Autographiviridae*; *Corkvirinae*; *Phimunavirus*; *Pectobacterium* virus Koot
MK095204	*Pectobacterium* phage Koot_B1	60,9	*Autographiviridae*; *Corkvirinae*; *Phimunavirus*; unclassified *Phimunavirus*
MK095210	*Pectobacterium* phage Zenivior	60,9	*Autographiviridae*; *Corkvirinae*; *Phimunavirus*; *Pectobacterium* virus Zenivior
MK095197	*Pectobacterium* phage Clickz_B5	60,8	*Autographiviridae*; *Corkvirinae*; *Phimunavirus*; unclassified *Phimunavirus*
MK095206	*Pectobacterium* phage Nobby_B2	60,8	*Autographiviridae*; *Corkvirinae*; *Phimunavirus*; unclassified *Phimunavirus*
KX237514	*Klebsiella* phage vB_KpnP_KpV48	60,7	*Autographiviridae*; *Slopekvirinae*; *Drulisvirus*; *Klebsiella* virus KpV48
KX397280	Phage MedPE-SWcel-C56	60,7	*Autographiviridae*; *Kafavirus*; *Kawavirus* SWcelC56
LC487410	*Vibrio* phage KIT04	60,7	*Demerecviridae*; *Ermolyevavirinae*; *Cetovirus*; unclassified *Cetovirus*
MK095211	*Pectobacterium* phage Zenivior_B1	60,6	*Autographiviridae*; *Corkvirinae*; Phimunavirus; unclassified *Phimunavirus*
MN794000	*Klebsiella* phage VLC1	60,6	*Autographiviridae*; *Slopekvirinae*; *Drulisvirus*; unclassified *Drulisvirus*
MT197176	*Klebsiella* phage VLC6	60,6	*Autographiviridae*; *Slopekvirinae*; *Drulisvirus*; unclassified *Drulisvirus*
JQ067087	*Pseudomonas* phage PaMx11	60,5	*Siphoviridae*; *Abidjanvirus*

KT240186	*Dickeya* phage BF25/12	60,5	*Autographiviridae*; *Corkvirinae*; *Stompvirus*; *Dickeya* virus BF25-12
MN966731	*Vibrio* phage Chazly21	60,5	*Demerecviridae*; *Ermolyevavirinae*; *Cetovirus*; unclassified *Cetovirus*
MW344056	*Vibrio* phage ABurr	60,5	*Demerecviridae*; *Ermolyevavirinae*; *Cetovirus*; unclassified *Cetovirus*
HE956707	*Yersinia* phage phiR8-01	60,4	*Autographiviridae*; *Melnykvirinae*; *Pienvirus*; *Yersinia* virus R8-01
MN966730	*Vibrio* phage Cilsick	60,4	*Demerecviridae*; *Ermolyevavirinae*; *Cetovirus*; unclassified *Cetovirus*
MN966732	*Vibrio* phage Chester	60,4	*Demerecviridae*; *Ermolyevavirinae*; *Cetovirus*; unclassified *Cetovirus*
MT118296	*Pseudomonas* phage Epa19	60,4	*Siphoviridae*
GQ413938	*Klebsiella* phage KP34	60,3	*Autographiviridae*; *Slopekvirinae*; *Drulisvirus*
KX587949	*Klebsiella* phage phiKpS2	60,2	*Autographiviridae*; *Slopekvirinae*; *Drulisvirus*; *Klebsiella* virus KpS2
MK095201	*Pectobacterium* phage Ekidair	60,2	*Autographiviridae*; Corkvirinae; Phimunavirus; unclassified Phimunavirus
MN101219	*Klebsiella* phage KOX6	60,2	*Autographiviridae*; *Slopekvirinae*; *Drulisvirus*; unclassified *Drulisvirus*
MN101220	*Klebsiella* phage KOX7	60,2	*Autographiviridae*; *Slopekvirinae*; *Drulisvirus*; unclassified *Drulisvirus*
MN794001	*Klebsiella* phage VLC2	60,2	*Autographiviridae*; *Slopekvirinae*; *Drulisvirus*; unclassified *Drulisvirus*
MN794003	*Klebsiella* phage VLC4	60,2	*Autographiviridae*; *Slopekvirinae*; *Drulisvirus*; unclassified *Drulisvirus*
MK095194	*Pectobacterium* phage Clickz_B2	59,9	*Autographiviridae*; *Corkvirinae*; *Phimunavirus*; unclassified *Phimunavirus*
MT259468	*Aeromonas* phage PS	59,9	*Autographiviridae*; unclassified *Autographiviridae*
KY684082	*Shigella* phage SFN6B	59,8	*Autographiviridae*; *Slopekvirinae*; *Drulisvirus*; *Shigella* virus SFN6B
MG649966	*Vibrio* phage Ceto	59,8	*Demerecviridae*; *Ermolyevavirinae*; *Cetovirus*
MK693005	*Klebsiella* phage KPR2	59,8	*Autographiviridae*; *Slopekvirinae*; *Drulisvirus*; unclassified *Drulisvirus*
MT682065	*Klebsiella* virus KpV2883	59,8	*Autographiviridae*; *Slopekvirinae*; *Drulisvirus*; unclassified *Drulisvirus*
MT701589	*Klebsiella* phage Pone	59,8	*Autographiviridae*; *Slopekvirinae*; *Drulisvirus*; unclassified *Drulisvirus*
MW358930	*Providencia* phage PSTRCR_114	59,725	*Autographiviridae*; *Kakivirus*
KT367886	*Klebsiella* phage Kp2	59,7	*Autographiviridae*; *Slopekvirinae*; *Drulisvirus*
KY000081	*Klebsiella* phage KPV811	59,7	*Autographiviridae*; *Slopekvirinae*; *Drulisvirus*; *Klebsiella* virus KPV811
MK095195	*Pectobacterium* phage Clickz_B3	59,7	*Autographiviridae*; *Corkvirinae*; *Phimunavirus*; unclassified *Phimunavirus*
MN101217	*Klebsiella* phage KOX4	59,7	*Autographiviridae*; *Slopekvirinae*; *Drulisvirus*; unclassified *Drulisvirus*
MN380459	*Klebsiella* phage vB_KpnP_fHeKpn01	59,7	*Autographiviridae*; *Slopekvirinae*; *Drulisvirus*; unclassified *Drulisvirus*
KT345706	*Vibrio* phage vB_VorS-PVo5	59,6	*Demerecviridae*; *Ermolyevavirinae*; *Cetovirus*; unclassified *Cetovirus*
MK838107	*Aeromonas* phage LAh1	59,5	*Autographiviridae*; *Melnykvirinae*; *Ahphunavirus*; unclassified *Ahphunavirus*
MK838108	*Aeromonas* phage LAh2	59,5	*Autographiviridae*; *Melnykvirinae Melnykvirinae*; *Ahphunavirus*; unclassified *Ahphunavirus*
MK838109	*Aeromonas* phage LAh3	59,5	*Autographiviridae*; Melnykvirinae; *Ahphunavirus*; unclassified

			Ahphunavirus
MK838110	*Aeromonas* phage LAh4	59,5	*Autographiviridae*; *Melnykvirinae*; *Ahphunavirus*; unclassified *Ahphunavirus*
MK838111	*Aeromonas* phage LAh5	59,5	*Autographiviridae*; *Melnykvirinae*; *Ahphunavirus*; unclassified *Ahphunavirus*
MT197175	*Klebsiella* phage VLC5	59,5	*Autographiviridae*; *Slopekvirinae*; *Drulisvirus*; unclassified *Drulisvirus*
MK387869	*Providencia* phage vB PstP PS3	59,25	*Autographiviridae*; *Kakivirus*; *Providencia* virus PS3
KU666550	*Klebsiella* phage KpV71	59,2	*Autographiviridae*; *Slopekvirinae*; *Drulisvirus*
MN176573	*Klebsiella* phage vB_KpnP_IME337	59,2	*Autographiviridae*; *Slopekvirinae*; *Drulisvirus*; unclassified *Drulisvirus*
MT939252	*Klebsiella* phage vB_KpnP_Dlv622	59,2	*Autographiviridae*; *Slopekvirinae*; *Drulisvirus*; unclassified *Drulisvirus*
KF319020	*Proteus* phage PM16	59	*Autographiviridae*; *Slopekvirinae*; *Novosibovirus*; *Proteus* virus PM16
LC375533	*Pectobacterium* phage PPWS2	58,8	*Autographiviridae*; *Corkvirinae*; *Kotilavirus*; *Pectobacterium* virus PPWS2
MT460517	*Vibrio* phage Athena	58,8	*Demerecviridae*; *Ermolyevavirinae*; Cetovirus; unclassified Cetovirus
MK962640	*Achromobacter* phage vB_AxyP_19-32_Axy23	58,7	*Autographiviridae*; unclassified *Autographiviridae*
JQ340389	*Vibrio* phage pVp-1	58,6	*Demerecviridae*; *Ermolyevavirinae*; *Vipunavirus*; *Vibrio* virus pVp1
MK895508	*Vibrio* phage Brizo	58,6	*Demerecviridae*; *Ermolyevavirinae*; *Cetovirus*; unclassified *Cetovirus*
MK907780	*Vibrio* phage Pontus	58,6	*Demerecviridae*; *Ermolyevavirinae*; *Cetovirus*; unclassified *Cetovirus*
MW344055	*Vibrio* phage GRLPWR	58,6	*Demerecviridae*; *Ermolyevavirinae*; *Cetovirus*; unclassified *Cetovirus*
MN958086	*Vibrio* phage Bennett	58,5	*Demerecviridae*; *Ermolyevavirinae*; *Cetovirus*; unclassified *Cetovirus*
MT448616	*Vibrio* phage BBMuffin	58,5	*Demerecviridae*; *Ermolyevavirinae*; *Cetovirus*; unclassified *Cetovirus*
MG649967	*Vibrio* phage Thalassa	58,4	*Demerecviridae*; *Ermolyevavirinae*; *Cetovirus*
MH179473	*Aeromonas* phage 25AhydR2PP	58,3	*Autographiviridae*; *Melnykvirinae*; *Aerosvirus*; *Aeromonas* virus 25AhydR2PP
MT108725	*Pseudomonas* phage Epa5	58,3	*Siphoviridae*
FN594518	*Pseudomonas* phage phi-2	58,2	*Autographiviridae*; *Krylovirinae*; *Tunggulviirus*; *Pseudomonas* virus f2
FR687252	*Pantoea* phage LIMElight	58,2	*Autographiviridae*; *Limelightvirus*
MN844877	*Sphaerotilus* phage vB_SnaP-R1	58	*Autographiviridae*; unclassified *Autographiviridae*
KU197013	*Xanthomonas* phage XAJ24	57,9	*Autographiviridae*; *Pradovirus*; *Xanthomonas* virus XAJ24
MK962628	*Achromobacter* phage vB_AxyP_19-32_Axy09	57,9	*Autographiviridae*; unclassified *Autographiviridae*
MW286266	*Pseudomonas* phage Bertil	57,9	*Autographiviridae*; unclassified *Autographiviridae*
MW286268	*Pseudomonas* phage Strit	57,9	*Autographiviridae*; unclassified *Autographiviridae*

MN399336	*Edwardsiella* phage vB_EtaM_ET-ABTNL-9	57,7	*Myoviridae*
MK962638	*Achromobacter* phage vB_AxyP_19-32_Axy21	57,6	*Autographiviridae*; unclassified *Autographiviridae*
MW286267	*Pseudomonas* phage Misse	57,5	*Autographiviridae*; unclassified *Autographiviridae*
MT460515	*Vibrio* phage Cody	57,3	*Demerecviridae*; *Ermolyevavirinae*; *Cetovirus*; unclassified *Cetovirus*
JN651747	*Aeromonas* phage phiAS7	57,1	*Autographiviridae*; *Melnykvirinae*; *Aerosvirus*; *Aeromonas* virus AS7
KR054031	*Pseudomonas* phage DL62	57,1	*Autographiviridae*; *Krylovirinae*; *Phikmvvirus*; *Pseudomonas* virus DL62
MH992513	*Aeromonas* phage ZPAH7	56,8	*Autographiviridae*; *Melnykvirinae*; *Aerosvirus*; *Aeromonas* virus ZPAH7
MK330684	*Aeromonas* phage ZPAH7B	56,8	*Autographiviridae*; *Melnykvirinae*; *Aerosvirus*; *Aeromonas* virus ZPAH7
MT104471	*Pseudomonas* phage MR8	56,8	*Autographiviridae*; *Krylovirinae*
MT104472	*Pseudomonas* phage MR12	56,8	*Autographiviridae*; *Krylovirinae*
MT104477	*Pseudomonas* phage MR18	56,8	*Autographiviridae*; *Krylovirinae*
MN871483	*Pseudomonas* phage PaSz-8_45_42k	56,7	*Autographiviridae*; *Krylovirinae*; *Phikmvvirus*; unclassified *Phikmvvirus*
EU056923	*Pseudomonas* phage PT5	56,6	*Autographiviridae*; *Krylovirinae*; *Phikmvvirus*; *Pseudomonas* virus PT5
JQ307386	*Pseudomonas* phage vB_Pae-TbilisiM32	56,6	*Autographiviridae*; *Krylovirinae*; *Phikmvvirus*; unclassified *Phikmvvirus*
KX711710	*Pseudomonas* phage vB_Pae-TbilisiM32	56,6	*Autographiviridae*; *Krylovirinae*; *Phikmvvirus*; unclassified *Phikmvvirus*
MK053931	*Pectobacterium* phage Arno160	56,6	*Autographiviridae*; *Melnykvirinae*; *Wanjuvirus*; *Pectobacterium* virus Arno160
MT104470	*Pseudomonas* phage MR7	56,6	*Autographiviridae*; *Krylovirinae*
MG592574	*Vibrio* phage 1.204.O._10N.222.46.F12	56,3	*Autographiviridae*; *Cyclitvirus*; *Vibrio* virus *Cyclit*
AM265639	*Pseudomonas* phage LKA1	56,1	*Autographiviridae*; *Krylovirinae*; *Stubburvirus*
EU236438	*Pseudomonas* phage PT2	56,1	*Autographiviridae*; *Krylovirinae*; *Phikmvvirus*; *Pseudomonas* virus PT2
KC969441	*Pseudomonas* phage MBL	56,1	*Autographiviridae*; *Krylovirinae*; *Phikmvvirus*; unclassified *Phikmvvirus*
MN553585	*Pseudomonas* phage 4phiC20-1	56,1	*Autographiviridae*; *Krylovirinae*; *Phikmvvirus*; unclassified *Phikmvvirus*
MN553587	*Pseudomonas* phage phiKMVC5-121	56,1	*Autographiviridae*; *Krylovirinae*; *Phikmvvirus*; unclassified *Phikmvvirus*
MN553589	*Pseudomonas* phage phiKMVC5-121	56,1	*Autographiviridae*; *Krylovirinae*; *Phikmvvirus*; unclassified *Phikmvvirus*
MN615699	*Pseudomonas* phage vB_PaeP_SPCG	56,1	*Autographiviridae*; *Krylovirinae*; *Phikmvvirus*; unclassified *Phikmvvirus*
KT381879	*Caulobacter* phage Percy	56	*Autographiviridae*; *Percyvirus*; *Caulobacter* virus Percy
MK903278	*Xanthomonas* phage Pagan	55,6	*Autographiviridae*; *Pradovirus*; unclassified *Pradovirus*
MH746814	*Acinetobacter* phage vB_AbaM_B09_Aci05	55,3	*Myoviridae*; *Saclayvirus*; *Acinetobacter* virus Aci05
MN855912	*Siphoviridae* sp.	55,2	*Siphoviridae*

MK170160	*Acinetobacter* phage TAC1	55,1	*Myoviridae*; *Saclayvirus*; unclassified *Saclayvirus*
MT623546	*Acinetobacter* phage Ab_121	53,9	*Myoviridae*; *Saclayvirus*; unclassified *Saclayvirus*
